# CTCF As an Example of DNA-Binding Transcription Factors Containing Clusters of C2H2-Type Zinc Fingers

**DOI:** 10.32607/actanaturae.11206

**Published:** 2021

**Authors:** O. G. Maksimenko, D. V. Fursenko, E. V. Belova, P. G. Georgiev

**Affiliations:** Institute of Gene Biology RAS, Moscow, 119334 Russia; Center for Precision Genome Editing and Genetic Technologies for Biomedicine, Institute of Gene Biology RAS, Moscow, 119334 Russia

**Keywords:** C2H2-type zinc fingers, architectural proteins, transcription regulation, insulators, TAD, enhancers, promoters, CTCF

## Abstract

In mammals, most of the boundaries of topologically associating domains and all well-studied insulators are rich in binding sites for the CTCF protein. According to existing experimental data, CTCF is a key factor in the organization of the architecture of mammalian chromosomes. A characteristic feature of the CTCF is that the central part of the protein contains a cluster consisting of eleven domains of C2H2-type zinc fingers, five of which specifically bind to a long DNA sequence conserved in most animals. The class of transcription factors that carry a cluster of C2H2-type zinc fingers consisting of five or more domains (C2H2 proteins) is widely represented in all groups of animals. The functions of most C2H2 proteins still remain unknown. This review presents data on the structure and possible functions of these proteins, using the example of the vertebrate CTCF protein and several well- characterized C2H2 proteins in Drosophila and mammals.

## INTRODUCTION

Cell differentiation in higher eukaryotes has led to significant complication in the regulation of gene expression. Cell specialization is determined by transcription factor repertoires assembling on regulatory elements of the genome. The genes responsible for cell differentiation are usually regulated by enhancers, each of which activates a promoter in a particular group of cells for a specific time interval [1, 2, 3]. In some cases, transcription of the developmental genes is regulated by several dozens of enhancers; the distance between some of these enhancers and the regulated promoter can reach up to several hundred kilobase pairs.

The ability of enhancers to perform long-range stimulation of promoters has led to the assumption that there may be some specialized transcription domains within which contacts between enhancers and promoters occur more efficiently [4]. It was believed that at the boundaries of transcription domains there are special regulatory elements capable of blocking interactions between enhancers and promoters [5, 6]. The most common opinion was that domain boundaries interact either with each other or with the nuclear structures bound to the nuclear envelope. Indeed, regulatory elements with the predicted properties were found first in Drosophila and then in mammals; these elements became known as insulators [7]. The two main properties of insulators have been described using the model systems in transgenic Drosophila lines: their ability to block the enhancer–promoter contacts and that to prevent repression of transgene expression during its integration into the heterochromatin regions within the genome [5, 6].

The emergence of methods for genome-wide identification of contacts between chromatin regions *in vivo*, and high-resolution microscopy [8, 9, 10, 11], took the study of the spatial organization of the genome to a completely new level. It turns out that the chromosomes of all eukaryotes are organized into topologically associating domains (TADs), which are formed through predominant interaction between the ends or boundaries of the domains [12, 13, 14, 15]. In this case, contacts within a TAD form much more efficiently than contacts between sequences located in adjacent TADs.

The discovery of TADs gave grounds to assume that their boundaries correspond to the insulators that restrict independent regulatory domains [16, 17, 18]. However, studies carried out on single cells have shown that TAD boundaries form as a set of preferred contacts and are not strict physical barriers blocking any trans-interactions between regulatory elements located in different TADs [12, 14, 19, 20]. Most of the characterized insulators are located within the same TAD. The improvement in the resolution of contact maps within the TAD has led to the discovery of subdomains, which usually correspond to local contacts between regulatory elements [19].

## CTCF AS THE BEST STUDIED PROTEIN WITH A C2H2 ZINC FINGER CLUSTER IN MAMMALS

The vertebrate protein CTCF (CCCTC binding factor), which has been well-studied in humans and mice [21, 22], is expressed at all ontogenetic stages in all cell types and is required during embryogenesis. Depending on the context, CTCF can act as a transcriptional activator or repressor. It is involved in the inactivation of one of the X chromosomes in mammals, it regulates alternative splicing of pre-mRNA in some genes, controls imprinting, participates in recombination and repair, and is responsible for the activity of enhancers, promoters, and insulators. However, the key role played by vertebrate CTCF in the chromosomal architecture is what has been described most thoroughly [23, 24, 25]. Mammalian genomes contain from 40,000 to 80,000 CTCF binding sites, with over 5,000 sites being conserved in different species and cell lines [21, 26]. Approximately 50%, 15%, and 35% of the CTCF binding sites are located, respectively, in intergenic regions, near promoters, and within gene bodies (30% residing in introns and 5% residing in exons) [27]. Mammalian CTCF consists of non-structured terminal regions and eleven zinc fingers residing in the central part of the protein; the first ten zinc fingers are C2H2-type, and the last one is C2HC-type. It is worth noting that proteins containing one or, less frequently, several clusters of C2H2 zinc finger domains constitute a significant portion of all the C2H2 zinc finger proteins [28]. The classical C2H2 domain has the consensus sequence CX_2-4_CX_12_HX_2-8_H. In the presence of a zinc ion, this sequence folds to form a ββα structure, where zinc is tetrahedrally coordinated by two cysteine residues at one end of the β-sheet and two histidine residues at the C-ends of α-helices. The structure is stabilized by hydrophobic bonds. In the canonical complex, the α-helical sections of tandem C2H2 zinc fingers are located in the major groove of DNA. The high-affinity specific binding is ensured by specific interactions with nitrogenous bases and nonspecific contacts with the phosphate backbone of DNA. For any DNA triplet, it is possible to choose a C2H2 domain carrying the desired amino acids at key positions of the α-helix and specifically recognizing this triplet [29, 30, 31]. Therefore, just within a few years after the first description of the structure of the C2H2 domains bound to DNA, chimeric proteins consisting of a C2H2 domain cluster and the FokI domain introducing double-strand breaks in the DNA sequence started being actively used as site-specific endonucleases for targeted genome editing [32, 33].

**Fig. 1:**
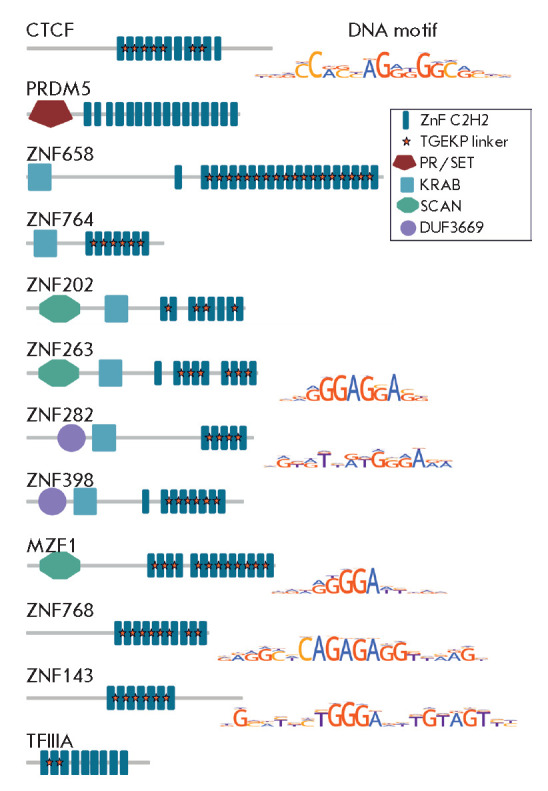
C2H2 proteins of vertebrates with architectural functions. The domain organization of the described proteins and the known binding motifs are shown

In proteins containing a C2H2-type zinc finger cluster, short 5-aa linkers residing between the domains possess the consensus sequence TGEKP and are a characteristic feature of DNA-binding C2H2 proteins [34]. The linkers are critical in terms of the affinity and specificity of DNA binding; mutations in them may cause a loss of the protein’s function *in vivo* [35, 36]. It is believed that each amino acid residue within the linker plays its own role in the interaction with DNA. Flexible in its unbound state, the protein structure, consisting of several C2H2 domains, “latches itself” as soon as it binds to the correct DNA sequence. The OH group of the first threonine residue T1 (or serine residue) forms a hydrogen bond with the amide group of glutamic acid E3; glycine G2 ensures the flexibility of the main chain required for latching. Glutamic acid E3 can contribute to the stabilization of the contacts between the zinc fingers. The lysine residue K4 (or arginine residue) is in contact with the DNA phosphate backbone. Proline residue P5 probably strengthens the bond between the linker and the following zinc finger; it also immobilizes the following conserved phenylalanine or tyrosine residue, whose aromatic ring lies on the N-end of the α-helix [37]. TGEKP-like linkers also connect the DNA-binding C2H2 domains of human CTCF *(Fig. 1)*.

The conformational changes in the DNA structure introduced by the C2H2 domains during binding limit the potential number of C2H2 domains connected by short linkers and capable of cooperatively interacting with DNA, and, therefore, limit the length of the canonical binding site [37]. This is probably why only four or five C2H2 domains are involved in the interaction and specific recognition of a 12–15 bp long DNA site in most proteins. Studies with artificial C2H2 clusters have shown that the specificity of protein binding to DNA increases when several short DNA-recognizing C2H2-domain clusters are connected by longer non-canonical linkers [28]. Therefore, it can be assumed that proteins carrying a large number of C2H2 domains in a cluster can specifically recognize different DNA sequences.

In human CTCF, the C2H2 domains **3**–**7 **are responsible for specific binding to the 15-bp consensus motif (*Fig. 1*) [38]. The C2H2 domain **8 **lies outside the major groove and is not involved in the recognition of DNA nitrogenous bases; therefore, it can act as a bridge connecting the C2H2-domains **3**–**7 **recognizing the key motif with the C2H2 domains **9**–**11**, which can specifically bind to an additional DNA motif that is found in approximately 15% of CTCF binding sites [39, 40]. The C2H2 domains **1**–**2 **can also bind to a non-conserved DNA sequence [39]. Thus, different combinations of C2H2 domains of CTCF can bind to a broad range of motifs with different levels of efficiency [41, 42].

It has been shown *in vitro *that CTCF–DNA binding is inhibited by cytosine methylation at position 2 in the consensus site, whereas cytosine methylation at position 12 has almost no effect. The cytosine at position 2 is recognized by the aspartic acid residue, which prefers the unmethylated base. At position 12, the cytosine is recognized by a glutamic acid residue, with the binding affinity slightly increasing in the case of a methylated base. [38]. Moreover, an important role in methyl group recognition is played by the arginine residue that forms the 5-methylcytosine–arginine– guanine triad in a complex with DNA; this triad is found in all C2H2 protein complexes with methylated DNA [43, 44].

Cytosine methylation at binding sites can enhance, weaken, or completely inhibit the binding of C2H2 proteins to DNA; i.e., it is a global mechanism of regulation of the promoter, enhancer, and insulator activities [45]. The most striking example of the role played by the methylation of binding sites for C2H2 proteins is the participation of CTCF in genomic imprinting, an epigenetic mechanism for regulating the expression of alleles of the same gene depending on their parental origin (male or female) [46]. Imprinting occurs with the participation of special regulatory elements known as differentially methylated regions (DMRs), which often contain CTCF binding sites. Imprinting has been described most thoroughly for the *Igf2 *and* H19 *genes, which are activated by a group of adjacent enhancers. The DMR acting as an insulator resides between the *Igf2 *gene and the enhancers; it consists of four CTCF binding sites carrying a cytosine residue at position 2. DMR methylation is maintained only in the paternal *Igf2*/*H19 *locus, resulting in a loss of CTCF binding and activation of the *Igf2 *gene. Meanwhile, in the maternal locus, CTCF binds to the corresponding sites in the DMR, thus inhibiting the interaction between the enhancers and the *Igf2 *gene. Methyla- tion of the binding sites for transcription factors (and C2H2 proteins in particular) can also be involved in the global inactivation of transcription in one of the two X chromosomes in mammals [47].

C2H2-domain clusters can participate in specific and non-specific interactions with RNA [48, 49]. Specific interaction between the TFIIIA protein and 5S RNA has been the one studied most thoroughly. It was shown that the C2H2 domains **1**–**3**, **5**, and **7**–**9 **bind to DNA motifs in the promoter region of the 5S RNA gene, while the C2H2 domains **4**, **5**, and **6 **interact with 5S RNA. Therefore, the C2H2 domains **4 **and **6 **act as linkers broadening the TFIIIA protein–DNA binding capacity. At the same time, specific interaction of these C2H2 domains with newly synthesized 5S RNA is necessary for its stabilization during export from the nucleus to the cytoplasm, prior to ribosome assembly.

Two C2H2 domains, **1 **and **10**, are responsible for the nonspecific interaction between CTCF and a broad range of RNAs [50, 51]. Interestingly, the disruption of the C2H2-domain structure caused by mutation in histidine does not affect RNA binding. This finding suggests that individual amino acids in the C2H2 domains play an important role in RNA binding, rather than the structure of the zinc finger itself. There are experimental data showing that interaction between CTCF and RNA may cause protein multimerization, but the mechanism of this process remains unknown [50, 52]. Since a large number of CTCF sites reside inside the introns of genes, it can be expected that CTCF is involved in the regulation of pre-mRNA splicing and termination (the processes running concomitantly with transcription) by non-specifically binding to RNA. For example, CTCF can slow down the movement of RNA polymerase II, leading to selection of either an alternative exon during splicing [53, 54] or an alternative polyadenylation signal during transcription termination [55]. A domain capable of interacting with RNA polymerase II has been mapped to the C-terminal domain of the CTCF protein, which can also be involved in the slowing-down of the movement of RNA polymerase II when passing through the CTCF binding sites [56].

A large body of experimental data shows that individual C2H2 domains or their clusters are involved in protein–protein interactions [34]. However, the detailed mechanisms behind these processes and their specificity remain poorly studied. C2H2 domains often interact with the complexes that are involved in chromatin remodeling and histone modification. According to data obtained through mutational analysis, any amino acids within the C2H2 domains and linkers connecting them can participate in these interactions (unlike during DNA binding). Therefore, it can be assumed that, in some cases, even the C2H2 domains associated with DNA can participate in the recruitment of regulatory complexes to chromatin.

A cluster of C2H2 domains is the only conserved part of the CTCF protein that shares high homology in most vertebrates, insects, and some nematodes [57, 58, 59]. The CTCF protein is not found in plants, yeast, or roundworms. The distribution of CTCF binding sites in the genome is also characterized by a certain degree of conservatism. In particular, CTCF binding sites are found at the boundaries of the regulatory domains of the homeotic genes in mammals, fish, and Drosophila [60, 61], where CTCF performs an insulator function and delimits the regions where enhancers residing in the adjacent domains perform their function [62-66]. It is worth mentioning that the CTCF binding sites are located in the repetitive elements of mammalian genomes, which could be the starting point for the evolutionary expansion of the CTCF binding sites in the intergenic regions where TAD boundaries are located [26, 67].

Despite the absence of homologous regions, the N-terminal domains of the CTCF proteins in nine animal species belonging to different classes are represented by unstructured homodimerization domains [68]. Deletion of the dimerized domain within Drosophila CTCF significantly reduces the functional activity of the mutant CTCF [69]. It was discovered in mouse embryonic stem cells that the N-terminal domain is involved in the specific binding of CTCF to the respective sites [70]. A YxF motif was found between the N-terminal homodimerization domain and the C2H2 cluster, which is necessary for interaction with the SA2–Scc1 cohesin subcomplex [71]. A similar motif was also found in the CTCF of other animal species. Therefore, although there is no significant homology, the N-terminal domains of the CTCF proteins in different species share characteristic structural features. A region interacted with the SA2 subunit of the cohesin complex was previously mapped on the C-end of the CTCF protein *in vitro *[72]; however, a more recent study has failed to confirm this finding [71].

The roles played by C2H2 proteins largely depend on the proteins with which they interact. More than 90 potential CTCF partner proteins have been identified [73, 74]. However, the mechanisms and specificity of these interactions remain disputable. Most protein– protein interactions are found within the cluster of C2H2 domains and in the unstructured C-terminus of the CTCF protein. Many different C2H2 proteins can potentially interact with the same protein complexes through the C2H2 domains. CTCF was shown to interact directly with the catalytic subunit Brg1 of the SWI/SNF chromatin remodeling complex [74] and the general transcription factor II-I (TFII-I) [75]. Therefore, the most probable function of CTCF in the promoter regions of actively transcribed genes is participation in the formation of open chromatin regions through the recruitment of the SWI / SNF complex, which increases the mobility of nucleosomes. CTCF can also be involved in stabilization on promoters of the TFIID complex (TFII-I being a part of this complex). When CTCF is inactivated, the expression level drops significantly only in the genes whose promoter regions contain CTCF binding sites [76]. Thus, one of the key functions of CTCF consists in organizing active promoters. Interestingly, like many other C2H2 proteins, CTCF contains regions enriched in proline and acidic amino acids, which is typical of the transcription activators recruiting transcription complexes to chromatin.

A domain interacting with DEAD box RNA helicases was identified in the C-terminus of CTCF [74, 77], which may be related to the potential significant participation of CTCF in the regulation of splicing and transcription termination. To perform these functions, the found interaction of CTCF with topoisomerase II (Top2) is probably also needed [78]. Top2 regulates chromatin topology by introducing ATP-dependent double-strand breaks into DNA. The Top2 protein has been found in approximately half of all CTCF binding sites [78]. Top2 activity is most often observed in the close vicinity of CTCF binding sites [79]. It is thought that Top2 is recruited to the open chromatin regions that form at CTCF sites, and that direct protein-protein interactions enhance this process. Possibly, CTCF helps recruit Top2 to the introns and 3’-ends of genes, which might be required during gene transcription.

The activity of C2H2 proteins is regulated by various post-translational modifications. Phosphorylation of C2H2 proteins at the linkers between the C2H2 domains, which occurs during mitosis and reduces the efficiency of protein binding to chromatin, has been studied quite thoroughly [80, 81, 82, 83]. C2H2 proteins can also undergo further modifications, such as ubiquitination, SUMOylation, and poly-ADP-ribosylation [84]. The ribosylation site resides at the N-terminus of CTCF [85]; this modification can affect protein dimerization and its binding to the cohesin complex. Poly-ADP-ribosylation affects the localization of the CTCF protein in nuclear compartments, chromatin binding, and transcription regulation [85, 86, 87]. Interestingly, the N-terminus of human CTCF interacts with the C-terminus of nucleophosmin 1 (NPM1), which can be responsible for CTCF localization within the cell [88]. Sites for covalent attachment of the SUMO protein through lysine were found in the C-terminal domains of the CTCF protein [89]. The Pc2 protein belonging to the Polycomb group of transcriptional repressors was identified as a SUMO E3 ligase for CTCF. Within cell nuclei, CTCF and Pc2 are found in bodies enriched in Polycomb-group proteins.

It is assumed that by interacting with various proteins and forming homopolymers, SUMO catalyzes the formation of dense intranuclear protein structures (bodies) that can perform many functions, including being a source of spare proteins during chromatin formation on newly synthesized DNA during replication [90, 91]. SUMOylation of CTCF on chromatin can also regulate the recruitment of transcriptional complexes to chromatin, thus changing the properties of CTCF during the activation or repression of gene transcription.

As a member of the C2H2 protein family, CTCF has typical structural features: it contains a cluster of zinc fingers that provides specific binding to genomic targets and interacts with RNA and proteins, as well as terminal domains that are involved in the organization of the architecture of chromosomes and the recruitment of various regulatory complexes to chromatin.

## CTCF IN ORGANIZATION OF THE CHROMOSOME ARCHITECTURE AND INSULATION IN VERTEBRATES

The CTCF protein was initially considered as the main vertebrate insulator protein [92]. The first vertebrate insulator was reported to be located at the boundary of the heterochromatin region and the chicken β-globin gene cluster [93, 94]. The insulator, with its core being 275-bp long, was mapped in the DNase 1 hypersensitive site and was therefore named HS4 [95]. In transgenic cell model systems, two copies of the HS4 insulator can effectively block enhancer activity and protect transgene expression from repression by surrounding chromatin. In addition to the binding site for the CTCF protein, the HS4 insulator was found to contain binding sites for USF1/USF2 proteins [96] and three binding sites for the VEZF1 (vascular endothelial zinc finger 1) protein [97]. It has been demonstrated that CTCF is required to block enhancers and recruit USF1/USF2 proteins, which in turn, recruit the complexes responsible for chromatin remodeling and histone modification. As a result, the nucleosomes around the HS4 insulator are enriched by nucleosome modifications associated with active chromatin (histone H3 methylated at lysine 4 and acetylated histones H3 and H4).

The VEZF1 protein contains a cluster consisting of six C2H2 domains, and it predominantly binds to active promoters [98]. Inactivation of the VEZF1 binding sites on the HS4 insulator in transgenic cell lines enhances DNA methylation at the promoter of a reporter gene [97]. It is assumed that VEZF1 recruits a complex that performs DNA demethylation, thereby facilitating the recruitment of transcription factors (which cannot efficiently bind to methylated sites) to the HS4 insulator and the adjacent regulatory elements. Thus, the HS4 insulator is a combination of the binding sites of at least two C2H2 proteins that function in close cooperation with each other.

Despite numerous examples illustrating the key role of CTCF sites in the organization of the boundaries of regulatory domains and the insulation of enhancers [23], the question remains as to what role is played by other unknown proteins whose binding to a particular regulatory element depends on the presence of CTCF. For example, in mammals, a large number of CTCF-dependent insulators block the spread of Polycomb-dependent heterochromatin, which is associated with H3K27me3 enrichment of long chromatin regions. However, inactivation of CTCF does not cause the propagation of the H3K27me3 modification into transcriptionally active regions, which suggests that other proteins are present at the boundaries that block the spreading of repressive chromatin and thereby mask the absence of CTCF [76]. Therefore, CTCF-dependent insulators, the boundaries of the regulatory domain, and TADs are most likely to consist of CTCF sites, in combination with the binding sites for other transcription factors (including C2H2 proteins that have not been described yet).

In our current understanding, which is supported by plenty of experimental data, mammalian CTCF forms chromatin loops, in cooperation with the cohesin complex, and defines the boundaries of most TADs [19, 99]. The cohesin complex is involved in mitosis, meiosis, and the regulation of gene expression [100, 101]. This complex consists of the SMC1, SMC3, and SCC1 (Rad21) proteins, forming a ring structure and binding to the fourth subunit that exists as two isoforms, STAG1 (SA1) and STAG2 (SA2), through SCC1. It has been hypothesized that SA1 and SA2 can determine the location of the cohesin complex at different chromatin sites. The NIPBL/MAU2 and WAPL complexes catalyze the ATP-dependent binding of the cohesin complex to chromatin and its subsequent dissociation, respectively [100].

Depending on the antibodies used and the cell line under study, the colocalization of CTCF and cohesin sites varies from 40% to 95% [102, 103, 104]. Inactivation of CTCF leads to a redistribution of the cohesin complexes from the CTCF binding sites to the promoters of active genes, accompanied by partial destruction of TADs [76]. Inactivation of the subunits of the cohesin complex or the Nipbl protein [105, 106], which ensures the recruitment of the cohesin complex to chromatin, leads to an almost complete disappearance of TADs. On the contrary, inactivation of the factors that negatively affect cohesin binding to chromatin stabilizes TADs and the long-range interactions in chromatin [106]. Finally, mutations and deletions in CTCF, disrupting its interaction with the cohesin complex, also significantly disturb the formation of long-range contacts and TADs [71, 104]. The Smc1 and Smc3 subunits contain ATPase domains, and the energy of ATP cleavage is required at the stages of binding and dissociation of the cohesin complex [107, 108]. Mutations in the subunits of the cohesin complex, which disrupt ATP hydrolysis, affect long-range contacts and the formation of TAD in chromosomes [109].

CTCF sites at the TAD boundaries usually have a convergent orientation [8, 110]. The convergent orientation of CTCF motifs was shown to define which pairs of CTCF sites preferentially stabilize DNA loops [8, 110, 111, 112]. A loop extrusion model has been proposed to explain why chromatin loops preferentially form between CTCF sites with a convergent orientation. According to this model, after being loaded onto chromatin, the cohesin complex triggers DNA extrusion and chromatin loop formation. CTCF can inhibit the movement of the cohesin complex only if its N-terminal domain, which interacts with the SA2–SCC1 subcomplex [71], is correctly oriented relative to the cohesin sliding complex.

The model postulates that the cohesin complex can induce chromatin extrusion and chromatin loop formation, either actively (using ATP energy) or passively. Indeed, *in vitro *studies have shown that in the presence of Nipbl and ATP molecules, the cohesin complex binds to DNA and slides along, causing loop formation [113], even if DNA is nucleosome-bound [114]. Cohesin can also bypass small nucleosome-sized protein complexes, but it is unable to overcome complexes larger than 13 nm in diameter; such complexes with a motor function can move cohesin themselves [115]. Therefore, the convergent CTCF sites limit the extrusion regions of chromatin loops, while the loop formation is performed by molecular motors.

According to the polymer model, TAD formation strongly depends on the physical properties of chromatin, which tends to form domains of the same type. This model has support in Drosophila, where TADs form through electrostatic inter-nucleosomal interactions. As a result, the TADs boundaries are predominantly composed of long, highly transcribed open chromatin regions and the inner regions of TADs are denser chromatin structures [13, 19, 116, 117]. In this model, the role of CTCF is to recruit cohesin complexes, stabilizing the interactions between chromatin sites that are already in close vicinity to each other. However, this model does not explain why chromatin loops in mammals predominantly form only convergently oriented CTCF binding sites.

The experimental data [107, 113, 118] show that the size of a chromatin loop is independent of the time of cohesin–DNA binding but depends on the barriers limiting its sliding (similar to CTCF). CTCF binds dynamically to chromatin, which is consistent with the heterogeneity of the TAD boundaries observed in studies of single cells [20]. The CTCF binding sites at the TAD boundaries are usually represented by clusters, which probably ensure CTCF binding to the genomic targets for a longer time [119].

According to the loop extrusion model, the cohesin complexes are only transiently blocked at a certain CTCF site and can continue to pull chromatin after crossing the block created by CTCF or after CTCF leaves chromatin [20]. Inactivation of Wapl stabilizes the binding of the cohesin complexes to chromatin; the size of chromatin loops increases, which is explained by longer residence time of the cohesin complex on chromatin [106, 120, 121].

Mitosis is characterized by chromosome condensation associated with large-scale chromatin changes and the loss of binding of some transcription factors to DNA. During mitotic prophase, most of the cohesin leaves the chromosomes (except for cohesin associated with centromeres). In anaphase, cohesin dissociation caused by separase promotes the segregation of sister chromatids [101]. The structure of TADs on compact mitotic chromosomes is lost almost completely but is quickly restored by the mid-G1 stage [122]. The data on the binding of CTCFs to the respective sites on mitotic chromosomes are inconsistent. According to some estimates, CTCF remains on 18.6% of the sites [122]; however, the binding of CTCF to its sites is mostly lost, since there is phosphorylation of linkers between the C2H2 domains [123]. It is possible that by leaving its binding sites, CTCF contributes to a more efficient removal of the cohesin complexes from mitotic chromosomes. However, CTCF binding sites are rapidly restored after mitosis, which may result from the association between free CTCF and condensed chromosomes during mitosis [123]. It remains an open question how the effective restoration of CTCF binding to the corresponding sites after mitosis occurs. It is most likely that other transcription factors remain associated with mitotic chromosomes and maintain the partially open chromatin state (act as bookmarks), which facilitates CTCF binding to the respective sites after mitosis. As a result, both the CTCF binding profile and the structure of TADs on duplicated chromosomes are rapidly restored after DNA replication. It can be assumed that excess CTCF accumulates in specialized nuclear compartments (bodies) stabilized by SUMO [89]. During replication, the excess CTCF binds to an increasing number of sites on duplicating chromosomes.

## ARCHITECTURAL FUNCTIONS OF OTHER VERTEBRATE C2H2 PROTEINS

Studies focused on designing artificial C2H2-type zinc fingers that ensure specific interaction with a particular genomic target have shown that the specificity of DNA binding increases dramatically for the cluster consisting of five properly organized zinc fingers. Therefore, in this section we would like to discuss proteins with this structural organization (with at least five C2H2-type domains separated by a typical 6-bp linker sequence) as the most promising architectural C2H2 proteins.

Other C2H2 proteins remain relatively less well studied than CTCF [124, 125]. The key problems in studying this class of proteins are associated with a significant overlap of functions between different C2H2 proteins and the lack of high-quality specific antibodies against these proteins, which would make it possible to perform genome-wide studies to identify the binding sites for C2H2 proteins and their role in the maintenance of long-range contacts between regulatory elements and the formation of the chromosome architecture. Two studies [126, 127] have focused on binding sites for the 60 and 221 C2H2 proteins with GFP or HA epitope tags in HEK293T cells. The binding sites for the same C2H2 proteins studied in both publications were found to overlap only slightly [128]. It should be noted that in these studies, expression of tagged C2H2 proteins occurred in the presence of the endogenous C2H2 proteins; so, most actual binding sites were occupied by the native protein, while the tagged protein was bound (mostly non-specifically) to the domains within open chromatin regions. In the near future, the use of the CRISPR/Cas9 genome editing tool could make it possible to replace endogenous genes with modified ones expressing tagged variants of C2H2 proteins, which will simplify genome-wide studies.

Mammalian genomes are enriched in various types of repetitive sequences of differing nature, including mobile elements and retroviruses [129]. Most of the studied C2H2 proteins, including CTCF, have binding sites in different repetitive sequences [130, 131, 132, 133]. There are many examples of repeating sequences becoming part of genetic regulatory networks and TAD boundaries [134], thereby significantly expanding the possibilities of fine-tuning gene expression during evolution.

Approximately half of all C2H2 proteins carry another domain at their N-terminus. The two most evolutionarily ancient domains that are found in all eukaryotes include the PR/SET domain (e.g., Prdm5 protein (*Fig. 1*)), which typically exhibits methyltransferase activity [135], and the BTB domain that forms dimers and recruits transcription regulators to the genomic targets [136]. One of the most numerous groups of C2H2 proteins in mammals carries the KRAB domain at their N-terminus (e.g., ZNF658 and ZNF764 proteins (*Fig. 1*)). It is believed that this domain has become widespread in mammalian C2H2 proteins, due to its repressor function with respect to mobile elements. However, in parallel with the evolution of gene regulatory systems into which mobile elements are integrated, KRAB-C2H2 proteins acquire new functions in the regulation of host gene expression [130, 131]. Some of these C2H2 proteins containing the KRAB domain carry an additional domain, SCAN (e.g., ZNF202 and ZNF263 proteins) or DUF3669 (e.g., ZNF282 and ZNF398 proteins), at their N-terminus [137, 138, 139]. Some C2H2 proteins carry only the SCAN domain (e.g., MZF1) and derive from proteins that have lost their KRAB domain. It can be assumed that some functions of the SCAN-C2H2 and DUF3669-C2H2 proteins are associated with the ability of SCAN and DUF3669 to form homo- and heterodimers [131, 137].

The most thoroughly described functions of C2H2 proteins are the formation of an open chromatin region on promoters and recruitment of transcription complexes for transcriptional activation or repression. The ZNF658 protein binds to the regulatory element residing next to 3525 promoters and participates in the activation of the expression of the rRNA genes transcribed by RNA polymerase I [140, 141]. The ZNF764 protein is ubiquitously expressed; it is involved in the regulation of glucocorticoid, androgen, and thyroid hormones activity [142]. It is interesting that the binding sites, which predominantly reside in intergenic regions (60%) and introns (31%), significantly (37%) co-localize with the binding sites for glucocorticoid receptors (GRs) [143]. It has been experimentally proved that the KRAB domain of the ZNF764 protein directly interacts with the LBD domain of a GR, suggesting that these proteins bind cooperatively to the regulatory regions. The protein-binding sites of ZNF202 [126, 144], ZNF263 [145], MZF1 [146], ZNF768 [133], and Prdm5 [147] are predominantly located in the promoter regions of genes, indicating that these factors may contribute to the activation or repression of transcription, and promoter architecture.

The N-terminus of ZNF768 (*Fig. 1*) contains 15 heptad repeats that are similar to the C-terminal domain of RNA polymerase II [133] and are presumably involved in the recruitment of the transcription elongation complex to promoters.

It has been demonstrated that MZF1 can form heterodimers with other SCAN-containing proteins (ZNF24, ZNF174, and ZNF202) through the SCAN domain [148, 149]. The ZNF282 and ZNF398 proteins form homo- and heterodimers through the DUF3669 domain [150] and can bind to the promoters in a combinatorial manner [126]. The Prdm5 protein contains an N-terminal PR/SET domain that has lost its methylation ability and is possibly involved in protein–protein interaction [151, 152].

The protein ZNF143 (*Fig. 1*), which is crucial to the embryonic development of mammals, has been characterized thoroughly [153]. Its central part contains a cluster consisting of seven C2H2 domains. The N-terminal domain contains three 15-aa repeats separated by 10-aa to 12-aa spacers [154]. The C-terminal domain is enriched in acidic amino acids, which is typical for transcriptional activators. The ZNF143 binding sites reside within a region of approximately 2,000 promoters regulated by RNA polymerases II and III [155, 156, 157, 158]. The functional activity of ZNF143 near the promoters is related to the formation of open chromatin regions and its involvement in the recruitment of transcription activation complexes [159, 160, 161]. The ZNF143 protein has two partially overlapping consensus binding sites with the same core CCCAGA sequence [155], which can be explained by the different contributions of individual C2H2 domains to the recognition of two site variants. Genome-wide studies have shown that the ZNF143 protein may be involved in the formation of chromatin loops between enhancers and promoters [155, 156, 162-164].

A relatively large percentage of the binding sites of the Prdm5 and ZNF143 proteins colocalize with CTCF [143, 152, 163]. The Prdm5 protein has been found in association with cohesin and CTCF [152]. It was shown for HEK293T cells that inactivation of ZNF143 disrupts some CTCF-dependent chromatin loops [163]. However, there are no experimental data proving that ZNF143 (unlike CTCF) can be involved in the localization of the cohesin complex on chromatin.

Another example of the structural function of C2H2 proteins was observed when studying the chromatin architecture organized by the TFIIIC complex. It was found that the binding sites of the TFIIIC complex colocalize with condensins and can act as boundaries between active chromatin and heterochromatin, as well as maintain distant interactions and participate in the formation of the chromosome architecture [165]. Interestingly, the binding sites for Prdm5, CTCF/ cohesin, and ZNF143 proteins are adjacent to or colocalize with the TFIIIC binding regions [152, 155, 166], suggesting that these proteins are cooperatively involved in the organization of TFIIIC-dependent regulatory elements. Furthermore, the Prdm5 protein has been isolated in complex with TFIIIC, suggesting that Prdm5 participates in the recruitment of the TFIIIC complex to chromatin [152].

The above-mentioned TFIIIA is the second (after CTCF) best-studied C2H2 protein, which binds to Pol III-dependent promoters of the genes encoding 5S rRNA in all eukaryotes [167]. TFIIIA consists of nine C2H2 domains and a C-terminal activation domain called TAS (Transcription Activating Signal). The protein binds to a regulatory element called ICR, which is located in the transcribed parts of the genes. A structural analysis revealed that the C2H2 domains** 1**–**3 **and **7**–**9 **bind to two regions (the C and A boxes) of the ICR element; the central C2H2 domains bind specifically to 5S RNA [167]. The lack of homology in the amino acid sequence of TFIIIA proteins from different species suggests a parallel evolution of the DNA sequences of the promoters, 5S rRNAs, and C2H2 domains, which are involved in the specific binding of DNA and RNA. The TFIIIA protein determines open chromatin on the promoter, while the TAS domain is involved in the recruitment and stable binding of the TFIIIB complex to the promoter [168].

The existing data show that many C2H2 proteins are involved in the formation of active promoters, as well as the recruitment of transcription factors and complexes to regulatory elements. It is obvious that many C2H2 proteins duplicate each other’s functions, which makes it difficult to prove their role in the global organization of the chromosome architecture and regulation of transcription.

## C2H2 PROTEINS IN DROSOPHILA: DIFFERENT STRUCTURES BUT SIMILAR PROPERTIES

**Fig. 2:**
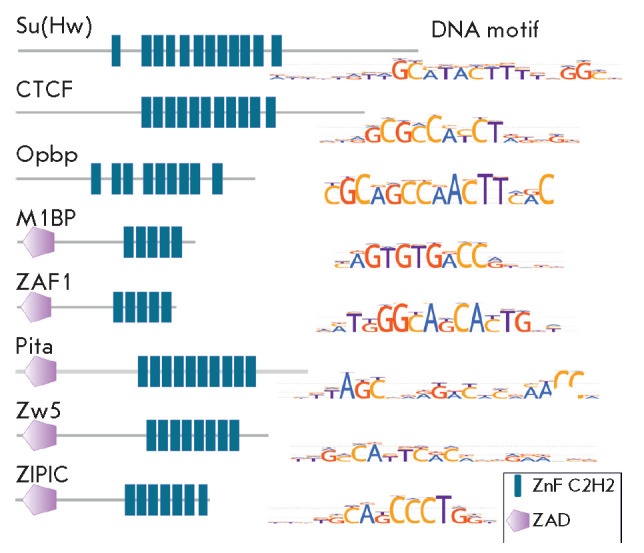
Drosophila C2H2 proteins with architectural functions. The domain organization of the known architectural proteins of Drosophila and their binding motifs are shown

Approximately 170 proteins with clusters consisting of at least five C2H2 domains have been found in the Drosophila genome. However, data on the distribution of C2H2 binding sites in the genome and their functional role in the regulation of gene transcription and the chromosomal architecture have been obtained for only a few of these proteins (*Fig. 2*). The best studied C2H2 proteins include the first protein with insulator properties described in higher eukaryotes, Su(Hw), and an homolog of mammalian CTCF, dCTCF [22, 24, 169, 170]. Both insulator proteins have a similar structure: they contain unstructured terminal domains and a central cluster consisting of 11 (dCTCF) or 12 (Su(Hw)) C2H2 domains. The N-terminus of the dCTCF protein contains an unstructured globular domain capable of forming tetrameric complexes [68, 69], and a potential site of interaction with the cohesin complex, which has homology with the human YxF motif of CTCF that interacts with the SA2-SCC1 complex [71]. An interesting structural feature of another studied C2H2 protein, Opbp [171], is the presence at an N-terminus of an atypical zinc finger capable of homodimerization (*Fig. 2*). Opbp has also a cluster consisting of five C2H2 domains responsible for specific binding to DNA and an additional four C2H2 domains that can interact with RNA and proteins.

The remaining five C2H2 proteins (M1BP, ZAF1, Pita, Zw5, and ZIPIC) belong to a large group of ZAD-containing proteins. The ZAD (zinc-finger-associated domain) was found in 98 Drosophila proteins; approximately 70 of these proteins contain five or more C2H2 domains [172, 173]. The genes encoding ZAD-C2H2 proteins are typically arranged in clusters and, like mammalian KRAB-C2H2 proteins [174, 175], actively evolve as a result of multiple duplications of the original gene copies. The ZAD structure is formed by two pairs of cysteine residues coordinated to the zinc ion [176]. The N-terminal portion of the domain is a globular structure; the C-terminal stem structure is formed by a long α-helix. ZAD domains are capable of homodimerization with the formation of an antiparallel dimmer [176, 177]. Mutations in the genes encoding the Pita and Zw5 proteins are lethal, which suggests an important role for some representatives of C2H2 proteins in the development of Drosophila [178, 179]. Inactivation of the Su(Hw) protein impairs gonad development, resulting in female sterility [180]. Like in mammals, the Drosophila CTCF protein is involved in the regulation of *hox *gene expression [181, 182]. Although the Drosophila genome was found to contain only ~ 40 Opbp binding sites, inactivation of this protein causes pupal mortality [171].

All the investigated C2H2 proteins bind to long (12–15 bp) DNA motifs via four or five C2H2 domains organized as a cluster (*Fig. 2*) [171, 183-186]. Except for Su(Hw), the binding sites of C2H2 proteins predominantly reside in the promoter regions of active genes and introns [177, 184-190]. The most illustrative example of a protein of this class is M1BP, which binds to the promoters of more than 2,000 genes [185] and, according to experimental data [191], participates in the formation of active promoters. The Opbp protein also binds exclusively to gene promoters, as it is colocalized with M1BP in about half of them [171]. Unlike M1BP and Opbp, which are involved in transcription activation, Su(Hw) binds to the promoters of a large group of neuronal genes and represses their transcription in female gonads during the early stages of Drosophila development [192, 193].

The role played by C2H2 proteins in the formation of long-range contacts and inhibiting enhancer activity was analyzed in transgenic Drosophila lines. *In vivo*, C2H2 proteins efficiently interact with artificial DNA fragments, each containing four to five binding sites [177, 184, 188, 194]. In transgenic lines, the activity of an enhancer surrounded by binding sites for the same C2H2 protein is substantially blocked. However, the enhancer activity is restored by the removal of either of the two binding sites of the C2H2 protein, which proves that the interaction between the C2H2 proteins plays a crucial role in the formation of the chromatin loop, resulting in steric isolation of the enhancer. In the transgenic model system, the C2H2 protein binding sites can bring the yeast GAL4 activator and the reporter gene promoter closer together, thus activating transcription [177, 184, 195]. At the same time, combinations of binding sites for different C2H2 proteins cannot bring the GAL4 activator closer to the promoter [177, 195], which can explain the importance of preferential homodimerization of C2H2 proteins in providing specific distant interactions between genomic elements. For example, it has been shown that the ability of the ZAF1 and ZIPIC proteins to maintain distant interactions is determined by their ZAD domains [177, 184]. Therefore, domains capable of forming homodimers seem to play an important role in the organization of specific longrange contacts between the regulatory elements in chromatin.

The role of C2H2 proteins in the organization of the boundaries of regulatory domains can be most clearly demonstrated by the example of the bithorax complex (BX-C), which includes three homeotic genes, Ubx, abd-A, and Abd-B [196, 197]. The regulatory region of the BX-C is divided into nine independent domains, each activating the transcription of one of the three homeotic genes during the development. Several domain boundaries are characterized in detail and mapped as minimal fragments that can function as effective insulators in transgenic model systems [198-202]. Each characterized boundary contains different combinations of the binding sites of the Pita, dCTCF, and Su(Hw) proteins required to ensure its functional activity [65, 66]. The boundaries can be replaced with repeats consisting of four to five binding sites for each C2H2 protein. Thus, despite the differences in their structural organization, the Su(Hw), Pita, and dCTCF proteins have similar functions and work in cooperation in such processes as the organization of regulatory domain boundaries [66, 203, 204].

Unlike in mammals, the boundaries of most TADs in Drosophila coincide with clusters of housekeeping genes [205, 206]. Thus, the M1BP protein (whose binding sites reside in many promoters of housekeeping genes) is most often found at the TAD boundaries, while the binding sites for other characterized C2H2 proteins usually reside inside the TADs. In embryos and embryonic cell lines, the dCTCF protein, despite its cohesin-binding motif, is rarely found at the boundaries of TADs, while from 40% to 60% of dCTCF sites colocalize with cohesin complexes on chromatin [205, 206, 207]. Binding of the bulk of cohesin is observed in the open chromatin zones of actively transcribed promoters [208]; therefore, it cannot be ruled out that C2H2 proteins play a direct or an indirect role (organization of open chromatin regions) in the recruitment of cohesin complexes. Interestingly, most TAD boundaries in a BG3 cell culture derived from Drosophila neural tissues coincide with dCTCF binding sites [207]. Therefore, the TAD boundaries in Drosophila can be changed during cell differentiation.

It is most likely that the TAD boundaries are fixed due to interaction between the protein complexes flanking TADs. In addition, for Drosophila, the existence of a mechanism of TAD formation has been demonstrated, and it is due to the electrostatic internucleosomal interactions that make transcriptionally active sites act as TAD boundaries [116].

## CONCLUSIONS

At present, the C2H2 proteins of higher eukaryotes remain the least studied class of transcription factors. The well-studied mammalian CTCF protein provides general insight into the properties, partners, and functions of this class of transcription factors. CTCF is probably the ancestor of the entire class of C2H2 proteins, which in the course of evolution could acquire new domains and bind to new DNA sequences. In this context, it is interesting that CTCF in both Drosophila and mammals is involved in the organization of the boundaries of the transcriptional domains of homeotic genes. Drawing on existing information, it can be concluded that C2H2 proteins in mammals and Drosophila are often involved in the organization of active promoters. By interacting with nucleosome remodeling complexes, C2H2 proteins can form open chromatin and become simultaneously involved in the recruitment of major transcription factors to promoters. Many well-studied regulatory elements (promoters and insulators in particular) carry combinations of binding sites for C2H2 proteins which function cooperatively in their interaction with chromatin. Some C2H2 proteins, including CTCF, have been found to contain N-terminal homodimerization domains that may be involved in the organization of specific longrange contacts. The motif interacting with the cohesin complex has been identified only in the CTCF protein. However, C2H2 proteins can probably interact with other surfaces in cohesin and condensin complexes, which is consistent with the localization of these complexes on active promoters.

It is believed that different mechanisms are responsible for TAD boundary formation and long-range contacts in mammals and Drosophila. However, there still remains an open question as to whether the mammalian cohesin complex can cause intensive chromatin loop extrusion during the formation of TADs and long-range interactions between the regulatory elements. It is also unclear why other higher eukaryotes do not have a similar mechanism, although the cohesin complex is highly conserved. Interestingly, the genome of danio fish contains neither CTCF nor the cohesin complex at most TAD boundaries [209], despite the fact that CTCF in danio and humans shows 86% homology. On the other hand, CTCF is found at the TAD boundaries in Drosophila neural cells [207]. It can be assumed that the mechanisms of formation of TADs are actually much more universal than it appears at present. C2H2 proteins such as Prdm5 and ZNF143 can stabilize CTCF binding to mammalian TAD boundaries and be involved in long-range interactions. Drosophila C2H2 proteins, by binding in various combinations to insulators (for example, as part of BX-C), allow two identical copies of the insulator to maintain super-long-range interactions, which is similar to the formation of the boundaries of a new TAD. In mammals, the TAD boundaries usually contain the most evolutionarily conserved clusters of CTCF sites [119]. It can be assumed that at the early stages of vertebrate evolution, multiplied copies of one or several types of mobile elements containing CTCF binding sites, in combination with the sites of other C2H2 proteins, formed long-range interactions, and some of them have given rise to TADs. Therefore, despite the considerable progress achieved in studying the spatial organization of the genome and, in particular, the architectural role of CTCF, many questions remain unanswered due to the lack of data on the other participants necessary for the formation of the nucleus architecture.

## Abbreviations

kbp: kilobase pairs
TAD: topologically associating domain
CTCF: CCCTC-binding factor
C2H2: a domain consisting of two cysteine and two histidine residues coordinated to a zinc ion
